# ER predicts poor prognosis in male lung squamous cell cancer of stage IIIA-N2 disease after sequential adjuvant chemoradiotherapy

**DOI:** 10.3389/fonc.2023.1158104

**Published:** 2023-04-27

**Authors:** Xue Yang, Lili Wang, Xiangfeng Jin, Rongjian Xu, Zhuang Yu, Hongmei Li, Haijun Lu, Ning An

**Affiliations:** ^1^ Department of Medical Oncology, The Affiliated Hospital of Qingdao University, Qingdao, Shandong, China; ^2^ Department of Pathology, The Affiliated Hospital of Qingdao University, Qingdao, Shandong, China; ^3^ Department of Thoracic Surgery, The Affiliated Hospital of Qingdao University, Qingdao, Shandong, China; ^4^ Department of Radiation Oncology, The Affiliated Hospital of Qingdao University, Qingdao, Shandong, China

**Keywords:** postoperative radiotherapy, lung squamous cell cancer, stage IIIA-N2, survival, estrogen

## Abstract

**Introduction:**

The efficacy of postoperative radiotherapy (PORT) is still unclear in non-small cell lung cancer (NSCLC) patients with pIIIA-N2 disease. Estrogen receptor (ER) was proven significantly associated with poor clinical outcome of male lung squamous cell cancer (LUSC) after R0 resection in our previous study.

**Methods:**

A total of 124 male pIIIA-N2 LUSC patients who completed four cycles of adjuvant chemotherapy and PORT after complete resection were eligible for enrollment in this study from October 2016 to December 2021. ER expression was evaluated using immunohistochemistry assay.

**Results:**

The median follow-up was 29.7 months. Among 124 patients, 46 (37.1%) were ER positive (stained tumor cells≥1%), and the rest 78 (62.9%) were ER negative. Eleven clinical factors considered in this study were well balanced between ER+ and ER- groups. ER expression significantly predicted a poor prognosis in disease-free survival (DFS, HR=2.507; 95% CI: 1.629-3.857; log-rank *p*=1.60×10^-5^). The 3-year DFS rates were 37.8% with ER- *vs.* 5.7% with ER+, with median DFS 25.9 *vs*. 12.6 months, respectively. The significant prognostic advantage in ER- patients was also observed in overall survival (OS), local recurrence free survival (LRFS), and distant metastasis free survival (DMFS). The 3-year OS rates were 59.7% with ER- *vs*. 48.2% with ER+ (HR, 1.859; 95% CI: 1.132-3.053; log-rank *p*=0.013), the 3-year LRFS rates were 44.1% *vs*. 15.3% (HR=2.616; 95% CI: 1.685-4.061; log-rank *p*=8.80×10^-6^), and the 3-year DMFS rates were 45.3% *vs*. 31.8% (HR=1.628; 95% CI: 1.019-2.601; log-rank *p*=0.039). Cox regression analyses indicated that ER status was the only significant factor for DFS (*p*=2.940×10^-5^), OS (*p*=0.014), LRFS (*p*=1.825×10^-5^) and DMFS (*p*=0.041) among other 11 clinical factors.

**Conclusions:**

PORT might be more beneficial for ER negative LUSCs in male, and the examination of ER status might be helpful in identifying patients suitable for PORT.

## Introduction

Radical surgery and dissection of mediastinal lymph node is the standard therapy for non-small cell lung cancer (NSCLC) patients with resectable lymph node(s) if the operation is endurable. Multiple randomized clinical trials (RCTs) confirmed a definitive survival benefit brought by adjuvant chemotherapy in selected patients (1–3). Nevertheless, disease-free survival (DFS) is still suboptimal, with considerable local failures leading to high risk in disease recurrence and worse overall survival (OS), especially in stage III N2 patients, even after adjuvant chemotherapy (4).

However, the evidences for postoperative radiation therapy (PORT) of R0 resected NSCLC are quite controversial. PORT has been found to be detrimental for pathologic N0/1 disease based on OS in meta-analyses (majorly population-based analysis of data from SEER database of small RCTs) (5, 6). Some meta-analyses showed a prognostic advantage of PORT in patients with pathologic N2 disease (6–8). However, the evidences from these meta-analyses were highly flawed. Most of these enrolled researches were from 1960s, when no definite staging system had ever been established. Moreover, the majority of patients received outdated radiotherapy technologies, for instance, 2-dimension conventional radiotherapy and Cobalt-60 equipment, leading to enormous unevenness in dose distribution and great heterogeneity in dose prescriptions, target volumes, and fractionations. Additionally, clinical information, including margin status, performance status, use of adjuvant chemotherapy and subsequent clinical implementations, was not available in these public databases, which was certainly not discussed in these meta-analyses. Besides, these analyses only took OS into consideration to evaluate the survival benefit brought by PORT, giving us no information about the DFS, local recurrence free survival (LRFS), and distant metastasis free survival (DMFS), which are also important to evaluate the therapeutic advantage of PORT after R0 resection.

Despite of some approval from meta-analyses, the therapeutic benefit of PORT in pIIIA-N2 patients was still unclear based on RCTs, especially in patients after R0 radical surgery and adjuvant chemotherapy. The ANITA retrospective RCT found that PORT increased OS in patients with pathologic N2 disease after adjuvant chemotherapy (9), whereas both LungART (10) and PORT-C (11) studies, the so-far only two completed prospective RCTs, failed in validating this survival advantage of PORT in stage IIIA-N2 patients. Therefore, the grim prospect of PORT in this subgroup of patients implies that a molecular predictor is urgently needed to identify the particular section of patients who can actually benefit from PORT.

Estrogen has been extensively reported to have an important function in NSCLC (12, 13). Some studies attempted to establish the correlation between estrogen receptor (ER) expression and NSCLC using immunohistochemistry (IHC) stain. Nevertheless, the reported results are contradictory and hard to interpret (14–17). Notably, the majority of these studies were only focusing upon female patients (18–20), probably caused by the stereotypical thinking that only women are subjected to the biofunction of estrogen. Moreover, the majority of ER-related studies in NSCLC were focusing on adenocarcinoma, while lung squamous cell cancer (LUSC) was seldom paid attention to let alone male LUSC patients. The treatment modality for lung adenocarcinoma has been ushered into a new era during the past decades. The tyrosine kinase inhibitors (TKIs) of EGFR and ALK have been proven remarkably beneficial in bringing a better clinical outcome in patients with lung adenocarcinoma in both adjuvant or salvage settings (21, 22), whose impact upon the observation of PORT efficacy was not considered by these RCTs. Thus, it is greatly necessary to analyze the efficacy of PORT in lung adenocarcinoma and LUSC separately, in order to eliminate the bias caused by targeted therapy.

LUSC patients are mainly male, and ER expression was reported as a significant unfavorable predictor of the clinical outcome in male LUSCs after radical resection in our previous study (23). In this study, we specifically focused on male stage IIIA-N2 LUSC who received sequential adjuvant chemotherapy and PORT, in attempt to establish the correlation between ER status and the prognosis of these patients. Despite the fact that the therapeutic effects of these inhibitors have been seldom discussed in LUSC patients (24), the EGFR mutation rate was reported around 5% in LUSCs, indicating these LUSCs might benefit from EGFR TKIs (25, 26). Therefore, in order to avoid the masking effect upon PORT by targeted therapy, molecular testing of EGFR mutation and ALK fusions was conducted in all the enrolled patients to exclude those with sensitive mutations of EGFR or ALK.

## Materials and methods

### Ethical approval

This study was approved by the Ethics Committee of the Affiliated Hospital of Qingdao University. Ethical standards of national and institutional research committee were strictly followed in all the procedures involving human participants. Written informed consent was provided by all the enrolled participants.

### Patient enrollment

Enrollment criteria in this study were as follows: male LUSC patients with the age 18 to 70 years old, weight loss < 10% before surgery, and Eastern Cooperative Oncology Group performance (ECOG) score < 2. Patients were excluded if they had any kind of neo-adjuvant treatments, a history of other cancer(s), EGFR sensitive mutation (including 19 exon deletion and 21 exon L858R mutation), ALK fusions, pneumonectomy, moderate/severe interstitial pulmonary disease, or uncontrolled infections. All the patients underwent thorough staging evaluations at most 60 days before surgery, including enhanced CT scan of the chest and abdomen; enhanced MRI of the brain; ultrasound test of supraclavicular lymph nodes and bone scan. Enrolled patients must be confirmed as pathologic stage IIIA-N2 (pT1-3N2) LUSC based on the seventh edition of American Joint Committee on Cancer staging system after R0 radical resection. Only those who completed the whole process of platinum-based adjuvant chemotherapy and PORT were enrolled.

### Surgery

After the diagnosis of LUSC through biopsy, patients were evaluated by a multiple disciplinary team (MDT), including at least a radiologist, a thoracic surgeon, a pathologist, a radiation oncologist, and a medical oncologist, to achieve consensus as follows: (a) technically resectable tumor. (b) N2 disease to the extent that adjuvant sequential chemoradiotherapy should be applied according to the knowledge at that time. All of the enrolled patients received lobectomy/bilobectomy of R0 resection, and complete dissection and exploration of the mediastinal lymph nodes, at least including the levels 4 (if accessible), 5, 6, 7, and 10 for left LUSC, and levels 4, 7, and 10 for right LUSC. All the resected lymph nodes were separately labeled with their corresponding locations for pathological examination. R0 resection was all confirmed by thoracic surgeons and two independent experienced pathologists.

### Sequential adjuvant chemoradiotherapy

Four cycles of platinum-based doublet regimen were administrated in adjuvant chemotherapy, i.e., GP [gemcitabine (1,000 mg/m2 intravenously on days 1 and 8) and cisplatin (40 mg/m2 intravenously on days 1-2) for every 21 days] or TP [paclitaxel (135 mg/m2 intravenously on days 1) and cisplatin (40 mg/m2 intravenously on days 1-2) for every 21 days]. Only intensity-modulated radiotherapy (IMRT) was adopted as the technique for PORT, with the clinical target volume (CTV) including the stump of the central lesions, the ipsilateral hilum, subcarinal region, and the region of bilateral mediastinum. The planning target volume (PTV) was formed by extending 0.5-0.8 cm margins from CTV (adjusted based on the irradiation and the condition of the residual lung). The total dose of radiation was up to 50 Gy at 2 Gy per fraction, 5 days per week, with 6 MV X-rays. Dose constraints for normal tissues were required as follows: the maximum dose should be ≤45 Gy for spinal cord; the mean dose should be ≤12Gy for lung, and ≤ 5% of the residual normal lung received 20Gy (V20 <25%); and the mean dose should be ≤30Gy for heart, with V30 < 40% and V40 <30%. PORT should proceed within six weeks from the fourth cycle of chemotherapy. The total interruption of PORT for any reason should be no more than 10 days.

### IHC assay to identify ER expression

The primary tumors embedded with formalin-fixed paraffin were collected. Slides of the tumors were stained with anti-ERα antibody (Zhongshan Bio-chemistry, China), and then incubated with anti-mouse secondary antibody (Zhongshan Bio-chemistry, China). The positivity of staining was evaluated based on PV-6000 detecting system, and each slide was then counterstained with hematoxylin. The microscope system of Olympus BX37 was adopted to obtain digital images. Each slide was examined by two blinded, experienced, and independent pathologists. The tumor was regarded as ER positive if IHC showed more than 1% of the cells were stained.

### Statistical analysis

DFS was defined as the duration from the date of operation to the date of any disease recurrence, death due to any cause or the last follow-up. OS was defined as the time span from the date of surgery to the date of death due to any cause or the last follow-up. LRFS was defined as the duration from the date of surgery to the date of loco-regional disease recurrence, death due to any cause or the last follow-up. DMFS was defined as the duration from the date of surgery to the date of distant metastasis of this disease, death due to any cause or the last follow-up. Common Terminology Criteria for Adverse Events (CTCAE, version 4.0) was adopted to grade the radiation toxicity related to PORT. R programming system (Version 4.0.3) was used in all the data analyses of this study. Log-rank test and Kaplan–Meier analysis were conducted to demonstrate the survival difference (significant if *p*<0.05). As for Cox analysis, variables of interest were first tested in univariate analysis, and those indicated as significant (if *p*<0.05) were further included in multivariate analysis to test their independence (significant if *p*<0.05). If only one variable was significant in univariate cox analysis, no further multivariate analysis was needed.

## Results

### Patient characteristics

**Figure 1:**
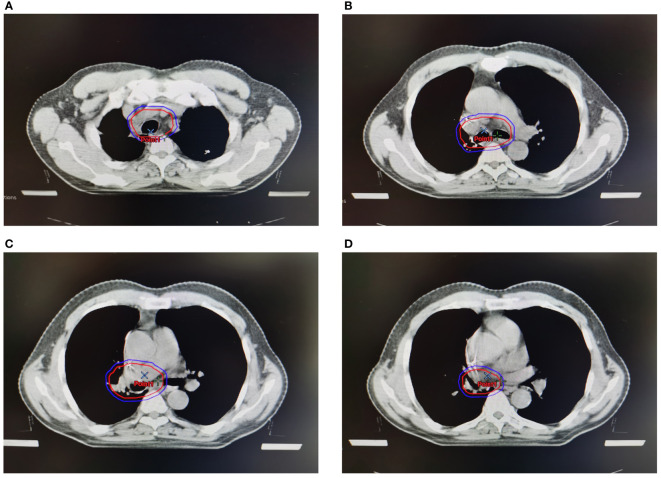
Clinical target volume (CTV) and planning target volume (PTV) of PORT. Red lines represented CTV, and blue lines represented PTV. **(A)**. CTV and PTV at the level of sternoclavicular joint. **(B)** CTV and PTV at the level of trachea carina. **(C)** CTV and PTV at the stump of the bronchia. **(D)** CTV and PTV at the level of ipsilateral hilum.

**Figure 2:**
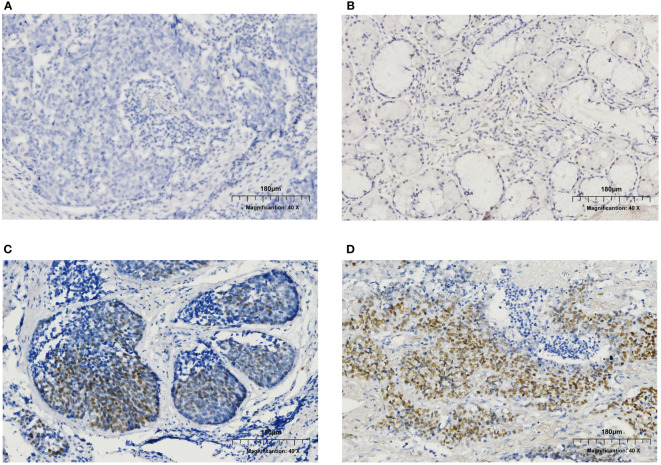
Immunohistochemistry (IHC) results of ER expression in male patients. **(A)** ER negative. **(B)** ER expression at the level of 5-10%. **(C)** ER expression at the level of 30-40%. **(D)** ER expression at the level of 70-80%.

In this study, 124 male LUSC patients who received complete resection in Department of Thoracic Surgery in the Affiliated Hospital of Qingdao University from October 2016 to December 2021 were enrolled based on aforementioned criteria. Clinical target volume (CTV) and planning target volume (PTV) were shown in **Figure 1**. The median follow-up time was 29.7 months, with the range from 3.4 to 65.3 months. Among these patients, 46 (37.1%) were ER positive, and the other 78 (62.9%) were ER negative according to IHC assay (**Table 1** and **Figure 2**). Eleven clinical factors were considered in baseline characteristic analysis, including age (<60 *vs*. ≥60 years old), ECOG score (0 *vs*. 1), grade (G1-2 *vs*. G3), pathological tumor size (pT, T1-2 *vs*. T3), visceral pleura invasion (positive *vs*. negative), vascular invasion (positive *vs.* negative), location (left *vs*. right), chemotherapy regimen (GP *vs*. TP), detected lymph nodes (DLNs, <20 *vs*. ≥20), positive N2 lymph nodes (PLNs, <3 *vs*. ≥3), and stations of N2 lymph nodes (<2 *vs*. ≥2). **Table 1** showed that all the clinico-pathological factors were well balanced between ER+ and ER- groups.

**Table 1:** Patient baseline characteristics.

Characteristics	ER+	ER-	*X* ^2^	*p*
Age (years)				
<60	12	25	0.248	0.619
≥60	34	53		
ECOG				
0	24	46	0.303	0.582
1	22	32		
Grade				
G1-2	22	34	0.074	0.786
G3	24	44		
pT				
T1-2	34	60	0.026	0.872
T3	12	18		
Visceral pleura				
Positive	12	25	0.248	0.619
Negative	34	53		
Vascular invasion				
Positive	27	37	1.053	0.305
Negative	19	41		
Location				
Left	24	35	0.360	0.548
Right	22	43		
Chemotherapy				
GP	24	30	1.691	0.194
TP	22	48		
DLNs				
<20	20	26	0.878	0.349
≥20	26	52		
PLNs				
<3	25	56	3.156	0.076
≥3	21	22		
Station				
<2	27	48	0.015	0.902
≥2	19	30		

DLNs, detected lymph nodes; PLNs, positive N2 lymph nodes.

### PORT toxicities

Acute toxicities related to PORT was defined as the adverse events happening within the duration between the beginning of PORT and the 3 months after PORT (**Table 2**). Twenty-two patients (17.7%) suffered from grade 1 (n=16) or grade 2 (n=6) acute pneumonitis. Additionally, 58 patients (46.8%) experienced grade 1 (n=48) or grade 2 acute esophagitis (n=10). No patients with grade 3 or higher acute pneumonitis or/and esophagitis were observed. There were 3 patients with grade 3 neutropenia and 5 patients with grade 3 thrombocytopenia. No patient with grade 4 or higher acute toxicities was observed (**Table 2**). There was no difference between two arms in respect to acute toxicities. As for late toxicity, only 4 patients (3.2%, 1 patient with ER+ and 3 with ER-) experienced pulmonary fibrosis. No treatment-related deaths have been observed for all the enrolled patients.

**Table 2:** Overall acute toxicities related to PORT.

Toxicity	ER positive (n=46)				ER negative (n=78)			
	Grade				Grade			
	1	2	3	4	1	2	3	4
Pneumonitis	6	2	0	0	10	4	0	0
Esophagitis	20	4	0	0	28	6	0	0
Neutropenia	6	5	1	0	10	9	2	0
Anemia	3	0	0	0	5	1	0	0
Leukopenia	5	2	0	0	9	4	0	0
Thrombocytopenia	5	3	2	0	8	5	3	0
Nausea and/or emesis	2	1	0	0	4	2	0	0
Cardiac	2	1	0	0	3	2	0	0
Fatigue	4	2	0	0	7	3	0	0

### ER expression predicted a poor prognosis

**Figure 3:**
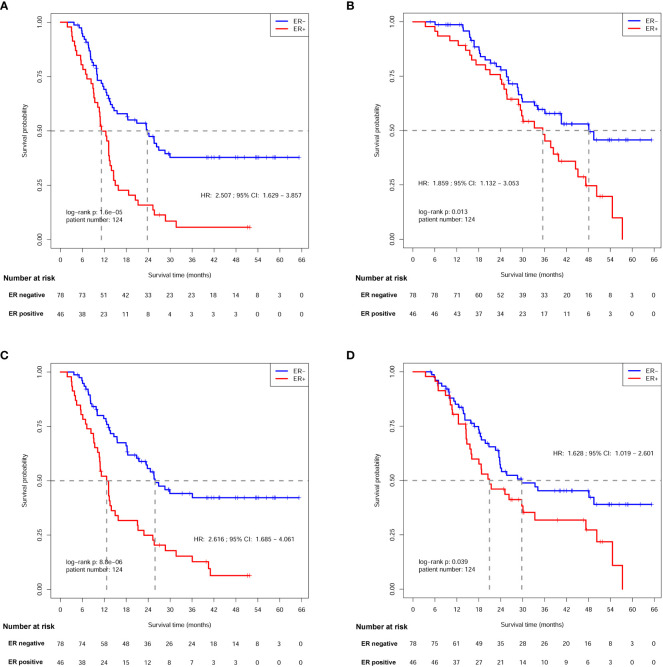
Survival analysis of patients between ER- and ER+ arms. **(A)** Disease free survival (DFS) analysis. **(B)** Overall survival (OS) analysis. **(C)** local recurrence free survival (LRFS) analysis. **(D)** Distant metastasis free survival (DMFS) analysis.

In this study, 86 DFS events (44 in ER- arm and 42 in ER+ arm) were observed at the time of the last follow-up of this study. The median DFS for ER- patients was 23.8 [95% confidence interval (CI), 14.6-NA] months, while the median DFS for ER+ arm was only 11.2 (95% CI, 10.2-13.9) months. The 3-year DFS for ER- and ER+ patients was 37.8% and 5.7%, respectively, showing a significant difference [hazard ratio (HR) = 2.507; 95% confidence interval (CI): 1.629-3.857; log-rank p=1.60×10^-5^; **Figure 3A** and **Table 3**].

**Table 3:** Cox regression analyses of DFS and OS.

Factors	DFS		OS	
	HR (95% *CI*)	*p*	HR (95% *CI*)	*p*
Age (years)				
<60	Reference	–	Reference	–
≥60	0.937 (0.603~1.456)	0.773	0.895(0.533~1.504)	0.676
ECOG				
0	Reference	–	Reference	–
1	1.086 (0.709~1.661)	0.705	1.122(0.680~1.853)	0.651
Grade				
G1-2	Reference	–	Reference	–
G3	1.085 (0.707~1.663)	0.709	1.153 (0.695~1.911)	0.582
pT				
T1-2	Reference	–	Reference	–
T3	0.972 (0.616~1.534)	0.903	1.067 (0.622~1.831)	0.814
Visceral pleura				
Negative	Reference	–	Reference	–
Positive	0.941 (0.591~1.499)	0.797	1.127 (0.666~1.907)	0.656
Vascular invasion				
Negative	Reference	–	Reference	–
Positive	1.219 (0.796~1.865)	0.362	1.406 (0.856~2.311)	0.179
Location				
Left	Reference	–	Reference	–
Right	0.946 (0.620~1.445)	0.798	0.751 (0.454~1.242)	0.264
Chemotherapy				
GP	Reference	–	Reference	–
TP	0.845 (0.553~1.291)	0.435	1.089 (0.661~1.793)	0.739
DLNs				
<20	Reference	–	Reference	–
≥20	1.099 (0.714~1.693)	0.667	0.907 (0.548~1.501)	0.704
PLNs				
<3	Reference	–	Reference	–
≥3	1.314 (0.850~2.030)	0.219	1.111 (0.666~1.854)	0.686
Station				
<2	Reference	–	Reference	–
≥2	0.965 (0.597~1.560)	0.245	0.877 (0.492~1.563)	0.295
ER				
Negative	Reference	–	Reference	–
Positive	2.507 (1.629~3.857)	**2.940×10^-5^**	1.859 (1.132~3.053)	**0.014**

Significant p values were in bold (p<0.05). HR, hazard ratio; CI, confidence interval.

Sixty-three deaths (31 in ER- arm and 32 in ER+ arm) were observed at the time of last follow-up. The median OS was 48.1 (95% CI: 34.1-NA) months for ER- patients, and 35.5 (95% CI, 28.9-45.1) months for ER+ patients. The 3-year OS rates were 59.7% and 48.2% for each arm, respectively, indicating a significant OS difference between patients with different ER status (HR, 1.859; 95% CI: 1.132-3.053; log-rank *p*=0.013; **Figure 3B** and **Table 3**).

Eighty-one patients (40 in ER- arm and 41 in ER+ arm) suffered from loco-regional recurrence. The median LRFS was 25.9 (95% CI: 21.5-NA) months in ER- patients, and the median LRFS was 12.6 (95% CI: 10.6-15.8) months for ER+ patients. The 3-year LRFS rates were 44.1% and 15.3% for ER- and ER+ arms, respectively, indicating a significant difference (HR=2.616; 95% CI: 1.685-4.061; log-rank *p*=8.80×10^-6^; **Figure 3C** and **Table 4**).

**Table 4:** Cox regression analyses of LRFS and DMFS.

Factors	LRFS		DMFS	
	HR (95% *CI*)	*p*	HR (95% *CI*)	*p*
Age (years)				
<60	Reference	–	Reference	–
≥60	1.031 (0.652~1.632)	0.896	0.915 (0.561~1.494)	0.722
ECOG				
0	Reference	–	Reference	–
1	1.154 (0.745~1.785)	0.521	1.157 (0.722~1.853)	0.545
Grade				
G1-2	Reference	–	Reference	–
G3	1.096 (0.705~1.705)	0.684	1.217 (0.755~1.959)	0.420
pT				
T1-2	Reference	–	Reference	–
T3	0.950 (0.592~1.523)	0.830	0.920 (0.551~1.535)	0.750
Visceral pleura				
Negative	Reference	–	Reference	–
Positive	0.935 (0.577~1.517)	0.787	0.926 (0.559~1.533)	0.764
Vascular invasion				
Negative	Reference	–	Reference	–
Positive	1.331 (0.858~2.064)	0.201	1.225 (0.767~1.956)	0.395
Location				
Left	Reference	–	Reference	–
Right	0.863 (0.558~1.335)	0.507	0.818 (0.511~1.309)	0.401
Chemotherapy				
GP	Reference	–	Reference	–
TP	0.753 (0.486~1.165)	0.203	1.474 (0.914~2.376)	0.112
DLNs				
<20	Reference	–	Reference	–
≥20	1.024 (0.657~1.597)	0.916	1.079 (0.670~1.736)	0.754
PLNs				
<3	Reference	–	Reference	–
≥3	1.304 (0.833~2.042)	0.246	1.262 (0.783~2.034)	0.339
Station				
<2	Reference	–	Reference	–
≥2	0.956 (0.584~1.565)	0.251	1.120 (0.645~1.944)	0.281
ER				
Negative	Reference	–	Reference	–
Positive	2.616 (1.685~4.061)	**1.825×10^-5^**	1.628 (1.019~2.601)	**0.041**

Significant p values were in bold (p<0.05). HR, hazard ratio; CI, confidence interval.

Seventy-one patients (38 in ER- arm and 33 in ER+ arm) suffered from distant metastasis. The median DMFS was 29.7 (95% CI: 23.5-NA) months in ER- patients, while the median DMFS was 20.9 (95% CI: 15.9-47.3) months in ER+ patients. The 3-year DMFS rates were 45.3% and 31.8% for ER- and ER+ arms, respectively, and a significant difference was observed between the two arms (HR=1.628; 95% CI: 1.019-2.601 log-rank *p*=0.039; **Figure 3D** and **Table 4**).

Cox regression analyses of DFS, OS, LRFS, and DMFS (**Tables 3**, **4**) were conducted among ER status and the other 11 clinico-pathological factors in these 124 patients, respectively. The result indicated that ER status was the only significant prognostic factor for DFS (*p*=2.940×10^-5^), OS (*p*=0.014), LRFS (*p*=1.825×10^-5^), and DMFS (*p*=0.041).

## Discussion

No concrete evidence has ever been established to support the prognostic advantage of PORT in pIIIA-N2 NSCLCs using modern radiotherapy techniques after R0 radical surgery and adjuvant chemotherapy, let alone for the subgroup of male LUSC patients. The landmark meta-analyses and RCTs were only concentrating on the clinical factors associated with the outcomes of PORT. However, the conflicting results of these studies demonstrated that only clinical factors were not sufficient to fulfill the mission, and molecular biomarkers should certainly be taken into consideration.

LUSC and lung adenocarcinoma, the two major components of NSCLC, were proven with great distinction on the basis of both pathology and treatment modality. Two milestone prospective RCTs, including ADAURA and EVIDENCE studies, demonstrated that EGFR TKIs could significantly improve clinical outcome and have a better tolerability profile in patients with EGFR-mutant NSCLCs after radical surgery (27, 28). Since almost all the EGFR-mutant NSCLCs were lung adenocarcinoma (more than 95% in ADAURA trial), EGFR TKI, instead of sequential chemoradiotherapy, was currently the standard of treatment for stage IIIA-N2 EGFR-mutant lung adenocarcinoma after complete resection. Therefore, LUSC and lung adenocarcinoma should be discussed separately in terms of adjuvant clinical implementations, and patients with driver gene mutations, including EGFR sensitive mutations or ALK fusions, were excluded from the present study in order to eliminate the systematic bias.

Although the optimal sequence of sequential adjuvant chemoradiotherapy is not established, PORT is generally administered after postoperative chemotherapy (29–31). Sequential adjuvant chemoradiotherapy in this study was strictly conducted according to PORT-C trial. Only those who completed all the four cycles of GP or TP chemotherapy and subsequent PORT of 2Gy×25 fractions were enrolled in attempt to decrease the potential bias causing by different clinical managements. Notably, only IMRT was adopted as the radiation technique to reduce the potential bias brought by other techniques, for instance, 3-dimensional conformal radiotherapy (3D-CRT) used by LungART and PORT-C studies. Modern radiation technology brings a very low toxicity, which hopefully might be translated into prognostic advantage of PORT. For instance, no grade 4 or higher adverse event related to PORT using IMRT has been observed in our study. The CTV in our radiation center includes the contralateral mediastinum but not supraclavicular region (**Figure 1**), of which the target volume is between PORT-C (ipsilateral mediastinum and subcarinal region) and LungART study (bilateral mediastinum and supraclavicular region). Superior 5-year OS advantage has been reported in N2 NSCLC patients who received PORT with the total dose between 45 to 54 Gy (32), while the prognostic advantage was not observed if the total dose > 54Gy because of an increased cardiac toxicity (33). Thus, all the enrolled patients received PORT with the dosage of 50Gy, in an attempt to balance between efficacy and toxicity. Both LungART and PORT-C studies failed in observing prognostic advantage of PORT in respect to DFS and OS, while both studies demonstrated the prognostic advantage of PORT in reducing local failure. It is possible since pIII-N2 NSCLC is highly heterogeneous, and thus only a part of patients could benefit from PORT.

The relationship between ER and NSCLC’s clinical outcome varies tremendously, and the most of these studies only focused on female adenocarcinoma. The remarkable controversy is probably due to many reasons, for instance, the patient population selected for research, the heterogeneous definitions of positivity, the differences in detecting methodology, and so on (14, 15, 34). Our previous finding indicated that the expression of ER predicted a poor clinical outcome in male LUSCs after receiving radical operation, which was also demonstrated by IHC assay (23). In present study, ER was significantly associated with DFS, OS, LRFS, and DMFS in male LUSCs after adjuvant sequential chemoradiotherapy. Currently, no effective biomarker has been confirmed to predict the therapeutic efficacy of PORT, and ER might be a promising biomarker to fulfill the mission. The result indicated that PORT might be more beneficial for ER negative LUSCs in male, and the examination of ER status might be helpful to identify male LUSCs suitable for PORT. As for ER positive male LUSCs with much worse prognosis, it is very intriguing that ER antagonist might be beneficial for treating these patients in adjuvant clinical setting.

The primary limitation of this study is the limited patient number (n=124), since we set a very strict enrollment criterion to reduce the potential bias. We only focused on male stage IIIA-N2 LUSCs with definitive molecular information of their EGFR and ALK status, and only patients strictly completed the sequential adjuvant chemoradiotherapy were enrolled, trying to validate the hypothesis inspired by our previous study (23). Additionally, this study is a single-center retrospective study. As we know, single-center studies have certain limitations in providing robustness and generalizability (35), but they might also reduce the bias brought by the inconsistency among different centers. However, external validations with more patients are certainly needed to further demonstrate the association between ER expression and PORT. PORT might be more beneficial for ER negative LUSCs in male, and the examination of ER status might be helpful in identifying patients with stage-IIIA N2 LUSC who are suitable for PORT.

## Data availability statement

The raw data supporting the conclusions of this article will be made available by the authors, without undue reservation.

## Ethics statement

The studies involving human participants were reviewed and approved by the ethics committee of the Affiliated Hospital of Qingdao University. The patients/participants provided their written informed consent to participate in this study.

## Author contributions

Data curation: LW, XJ, RX, HML, HJL, and ZY; formal analysis: NA and LW; funding acquisition: NA and XY; investigation: NA and XY; methodology: NA, XY, and LW; project administration: NA, XY, LW, XJ, and RX; resources: LW, XJ, RX, NA, and HJL; software: NA and LW; supervision: ZY; validation: XY; visualization: LW, XJ, and RX; writing-original draft: NA and XY; writing review and editing: all authors. All authors contributed to the article and approved the submitted version.
